# Advertising Ethics and Diabetes

**Published:** 1914-03

**Authors:** 


### Advertising Ethics and Diabetes

We acknowledge the valuable information given by the *Journal of the A. M. A* in its issue of Dec. 20, 1913, and at other times, regarding the actual composition of diabetic foods, and think that the time is ripe for emphasizing certain opinions regarding the general problem involved.

What is diabetes? Within recent years the attempt has been made to limit it to a definite disturbance of the Islands of Langerhans of the Pancreas, involving the lack of secretion of a glycolytic ferment. Unfortunately, this enticing theory is met by some very stubborn facts which seem to show that essentially typic cases of diabetes may occur independently of this lesion. It has even been questioned whether the conception of the insulae as minute ductless glands, differing essentially from the rest of the pancreas, is tenable. The dependence of diabetes or at least of glycosuria, upon disturbances of other ductless glands, notably the thyroid and adrenals, has been pretty well established, though not in an exclusive sense, and a very favorable tendency to connect well established physiologic knowledge with clinical medicine is evidenced by the recrudescence of interest in the glycogenic function or functions. It is very easy to say that mere glycosuria does not constitute diabetes, but it is very difficult to draw a practical line. There is a temptation to connect diabetes with acid intoxication and with disturbance of proteid metabolism. But there are cases which seem to warrant the term diabetes, in which there is no conspicuous, if any, increase of acetone bodies in general or any one of them, cases in which the urinary acidity is not notably increased, cases in which a high elimination of "urea" (which means practically non-proteid nitrogen in general) is either not noted at all or is pretty obviously dependent upon diet. Conversely, there are cases of acetonuria (implying acetonaemia) or of similar occurrence of its precursors, of high general urinary acidity and of high "urea" elimination which do not show marked similarity to ordinary cases of diabetes, not even justifying the French conception of a non-glycosuric diabetes. It is even observed that in cases of what may be considered true diabetes the reduction of sugar and of these other pathologic manifestations tend to occur synchronously or practically so.

There is obviously some physiologic function which enables the animal body to oxidize carbohydrates as ultimately digested into dextrose—or some other simple hexose?—since no such amount of oxidation can occur spontaneously, as normally occur in the body, under comparable conditions, unless some special glycolytic factor is conceded. Naturally, our first thought is of a ferment, and there is considerable evidence that such a ferment exists.

Clinically, the most practical classification of diabetic cases is between those that can and those that cannot be controlled by diet, etc., and this classification assumes more than clinical prognostic importance and implies a strictly scientific line of demarcation, when we reflect that the latter group imply the formation of sugar out of non-carbohydrate material. But it is impossible to draw lines between a mere excess of carbohydrate beyond the normal and more or less idiosyncratic glycolytic strength of the body and true diabetes of the first group, or to be certain that the two groups are absolutely distinct since the latter would imply that normal metabolism does not include sugar formation from non-carbohydrates even to a degree commensurate with glycolysis.

Now, let us define diabetes. . . . . Or, rather, let us confess that we are too ignorant to do so. If anyone wishes to show that "we" means the editorial and not the professional "we," he may supply the much desired definition. Of course, we do not allude to a definition that cannot be denied but which does not state the gist of the problem.

All things considered, one of the strongest if not the strongest weapon that we have against diabetes is dietetic regulation. Eliminative treatment, intestinal asepsis and even antisepsis, various other means empirically valuable though not fully explained are of considerable value. Direct sedation, as by codeine, we have almost never employed, and we doubt whether it is of any real value, except in the crudest symptomatic sense. A considerable degree of optimism is justifiable regarding diabetes. Cases of the second, extreme type, in which sugar is formed on a carbohydrate-free diet, are not very frequent, and the majority of cases may be kept in good general health, almost free from sugar in the urine, for years. We may cite a patient who, about seven years ago, passed almost exactly a pound of sugar in 24 hours, who has practiced medicine continuously, has not persisted in more than a moderate regulation of diet, who has indulged in carbohydrate and alcohol occasionally, without obvious impairment of health.

Regulation of diet implies a knowledge of what the diet contains. Herein is the ethical point first alluded to. A diabetic food should be not only relatively low in carbohydrates but it should be very decidedly so. At any rate, if sold especially for use by diabetics, exact statements of composition should be given.

If one will scrutinize the trite tables of what a dibetic may eat, in the line of vegetables, he will be impressed with two facts: most of the "foods" specified are not truly foods at all but fodder; of the "foods allowed" that do contain considerable proportions of starch, the preference over ordinary cereal and other foods rich in starch is due to the fact that the starch is not very digestible, or that the food produces intestinal digestion by hurrying peristalsis, or that the average patient will not care to eat much of the given food or some other equally superficial reason is apparent. Yeast has been advocated in the dietetic treatment of diabetes, but the reason is not difficult to find. By destroying the starch we have the same ultimate effect as if the patient had not eaten the food at all or only in small quantity but plus the discommoding action of the years on digestion generally. If it were mechanically possible it would be better to give the food with a literal string upon it, or to make the patient vomit after satisfying his appetite.

All things considered, a diabetic food means a harmless carbohydrate food or a food that appears to be carbohydrate but which is not. It is questionable whether one kind of starch is better than another or indeed whether, save for the necessary delay in digestion, starch is better than some form of sugar. The ordinary cereal foods contain somewhere about 50-75 per cent. of carbohydrates. It is difficult to conceive that a moderate reduction in the carbohydrate content has any practical value, certainly not unless the ration is accurately measured so as to prevent a compensatory appetite resulting in the patient's taking as much starch as he would in the interdicted foods. Why not administer the ordinary foods, up to the limit of tolerance, instead of allowing the patient to take more weight of a slightly reduced percentage content of carbohydræ, at several times the price of ordinary foods?

No objection can be made to allowing a variety of fodder vegetables. But we must realize that there is an appetite for carbohydrate which cannot be satisfied by any kind of deception of the palate. We may sweeten the patient's desserts and beverages with saccharin but we do not dispel the craving for real sugars—not to mention the toxic action of saccarin and the vileness of its after taste. Moreover, we must go farther than this. Acid intoxication can not be prevented unless some carbohydrate is given—somewhere about 8 grams a day, by empiric methods. Nor is the presence of sugar in the urine or in the blood essentially of dangerous import, save that in many cases sugar formation from non-carbohydrate material seems to be stimulated by even small amounts of carbohydrate ingestested. Carbohydrate may be entirely eliminated for a short period, without serious damage, but speaking generally, the diabetic who will be killed by carbohydrate in moderate or small amount, down to 80 grams a day, will die anyhow. We cannot get away from the administration of carbohydrates. To paraphrase the late President Lincoln, we can fool all of the carbohydrate appetites part of the time, that is to say, we can withdraw carbohydrates for a few days, occasionally, but it is doubtful whether we can fool any of them all of the time and, certainly, we can not fool the true, ultimate appetite for carbohydrates very long.

There is no further word to be said as to diabetic foods. Even from the standpoint of the palate, no satisfactory substitute for ordinary foods can be prepared without carbohydrates. Various kinds of crackers can be prepared from vegetable protein, casein, etc.; they can be given further variety by the incorporation of indigestible bean of different kinds, by artificial flavors; they can be rendered very agreeable to the person who samples them without need of a special food, but they cannot be prepared so as to form an adequate substitute for breadstuffs, etc. Except for the difficulty of marketing so as to insure freshness, diabetic foods can be manufactured which contain considerably less starch than ordinary cereal flours and breadstuffs. They are legitimate adjuvants to diet if honestly presented, with definite statements as to their contents. Even if sold with dishonest statements or with unwarranted generalization, they have a field of usefulness, if the medical profession is well enough informed to discount the advertisement. But, if used by a physician without discrimination or if employed by the laity directly, without caution, they are dangerous.

