# Corrections

**Published:** 1914-04

**Authors:** 


### EDITORIAL NOTE

Corrections. In the editorial on diabetes in the March issue, conflicting statements were printed as to the quantity of carbohydrate necessary to prevent acid intoxication. In each case the quantity should be stated as (approximately) 80 grams. We have never been able to ascertain whether this fact is purely empiric or whether it rests on some demonstrable physiologic principle. Perhaps some of our readers can give information.

The note in the Obituary Department the same month, regarding the liability to errors, was illustrated by the printer's putting an extra e in error.

